# ^1^H NMR-Based Metabolic Profiling Reveals the Effects of Fluoxetine on Lipid and Amino Acid Metabolism in Astrocytes

**DOI:** 10.3390/ijms16048490

**Published:** 2015-04-15

**Authors:** Shunjie Bai, Chanjuan Zhou, Pengfei Cheng, Yuying Fu, Liang Fang, Wen Huang, Jia Yu, Weihua Shao, Xinfa Wang, Meiling Liu, Jingjing Zhou, Peng Xie

**Affiliations:** 1 Department of Neurology, Yongchuan Hospital, Chongqing Medical University, Chongqing 402460, China; E-Mails: jasenbai@gmail.com (S.B.); yujialaowu2011@163.com (J.Y.); 2 Chongqing Key Laboratory of Neurobiology, Chongqing 400016, China; E-Mails: Veromcachou@gmail.com (C.Z.); cpfzero@163.com (P.C.); yingyuf0311@163.com (Y.F.); baisj888@126.com (L.F.); shaoweihua0615@aliyun.com (W.S.); lingyun155@126.com (X.W.); liumeilinglinsa@sina.com (M.L.); duduzjj@163.com (J.Z.); 3 Institute of Neuroscience and the Collaborative Innovation Center for Brain Science, Chongqing Medical University, Chongqing 400016, China; 4 Key Laboratory of Laboratory Medical Diagnostics of Education, Department of Laboratory Medicine, Chongqing Medical University, Chongqing 400016, China; 5 Department of Neurology, Xinqiao Hospital, Third Military Medical University, Chongqing 400016, China; E-Mail: huang_wen@163.com; 6 Department of Neurology, the First Affiliated Hospital of Chongqing Medical University, Chongqing 400016, China

**Keywords:** fluoxetine, SSRI, astrocytes, depression, metabolomics, ^1^H NMR

## Abstract

Fluoxetine, a selective serotonin reuptake inhibitor (SSRI), is a prescribed and effective antidepressant and generally used for the treatment of depression. Previous studies have revealed that the antidepressant mechanism of fluoxetine was related to astrocytes. However, the therapeutic mechanism underlying its mode of action in astrocytes remains largely unclear. In this study, primary astrocytes were exposed to 10 µM fluoxetine; 24 h post-treatment, a high-resolution proton nuclear magnetic resonance (^1^H NMR)-based metabolomic approach coupled with multivariate statistical analysis was used to characterize the metabolic variations of intracellular metabolites. The orthogonal partial least-squares discriminant analysis (OPLS-DA) score plots of the spectra demonstrated that the fluoxetine-treated astrocytes were significantly distinguished from the untreated controls. In total, 17 differential metabolites were identified to discriminate the two groups. These key metabolites were mainly involved in lipids, lipid metabolism-related molecules and amino acids. This is the first study to indicate that fluoxetine may exert antidepressant action by regulating the astrocyte’s lipid and amino acid metabolism. These findings should aid our understanding of the biological mechanisms underlying fluoxetine therapy.

## 1. Introduction

Major depressive disorder (depression) is a debilitating mental disorder affecting up to 15% of the general population and accounting for 12.3% of the global burden of disease [1,2]. Most antidepressants available today inhibit the reuptake or breakdown of serotonin and/or noradrenaline, of which SSRIs are the most common drug class [3,4]. Among SSRIs, fluoxetine has been approved for the treatment of depression since 1987 [5]; however, the cellular and molecular mechanisms underlying fluoxetine therapy remain largely unclear.

Astrocytes are major cells in the central nervous system (CNS) with numerous critical functions, and recent findings have reported their critical roles in neuronal development, neurotransmission and synaptic plasticity [6,7]. Meanwhile, mounting evidence has shown that astrocytes may be involved in the pathophysiology of depression. For example, astrocytic density and size are reduced in depression [8], and the loss of glial cells in the rat prefrontal cortex is sufficient to induce depressive-like behaviors in animal experiments [3]. Interestingly, fluoxetine prevents the stress-induced numerical decrease of astrocytes in the medial prefrontal cortex of rats subjected to chronic psychosocial stress [9]. Furthermore, many previous studies by our research group and others have revealed a strong relationship among lipid level, amino acid level, antidepressant and depression [2,10,11,12,13,14]. Thus, the observed astrocytic dysfunction in depression along with the reversal of stress-induced inhibition of gliogenesis by fluoxetine led us to hypothesize that fluoxetine may exert its antidepressant effects by regulating the astrocytes’ lipid and amino acid metabolism.

Recent work has indicated that fluoxetine could exert an antidepressant effort upon cortical astrocytes by regulating the expression of neurotrophic/growth factors [3]. However, little is known about the changes in the global metabolite expression in astrocytes treated with fluoxetine. Metabolomics, which includes NMR and gas/liquid chromatography-mass spectrometry (GC/LC-MS)-based metabolomic methods, have been widely used to investigate and profile metabolic variations of how antidepressants alter metabolic pathways in mammals [15,16,17,18]. Compared to GC/LC-MS, ^1^H NMR spectroscopy has been more popularly utilized, because it generally requires minimal sample preparation, presents high reproducibility, does not destroy the sample and has a short data acquisition duration [19]. Moreover, ^1^H NMR spectra generated from both hydrophilic and lipophilic sample extracts can improve the possibilities of a more detailed metabolite identification network and structural characterization.

In light of these facts, a ^1^H NMR-based metabolomic method was applied to characterize the intracellular metabolic profiles of astrocytes treated with fluoxetine *in vitro*. The objective here was to provide basic information about the underlying interaction of fluoxetine with astrocytes, to search for potential therapeutic targets for depression and to provide new directions for future studies on the biological mechanisms underlying fluoxetine exposure.

## 2. Results

### 2.1. Immunofluorescence Assay

**Figure 1:**
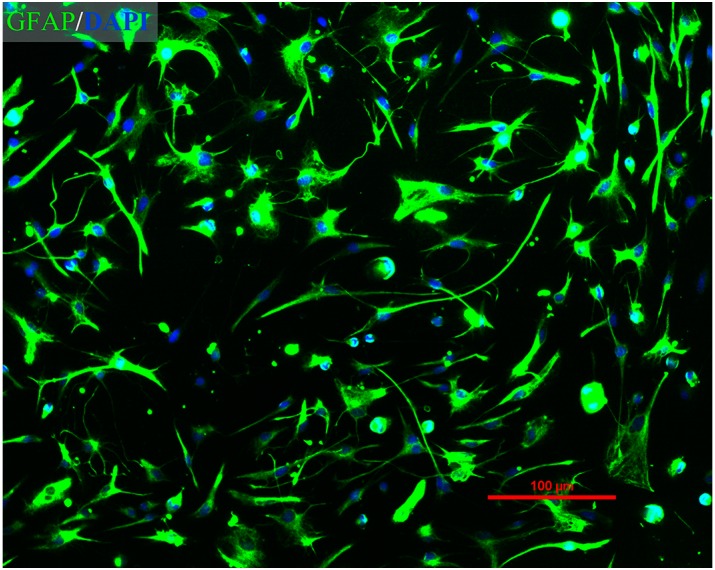
Immunofluorescence assay of purified primary rat astrocytes. Merged image of GFAP staining (green) and DAPI staining (blue) (magnification: 100×).

Immunofluorescent staining was performed to identify the purity of astrocytes. As shown in Figure 1, using this culture procedure, >95% of the cells were GFAP-positive. The percentage of labeled astrocytes was determined through observation of a minimum of 300 randomly-selected cells.

### 2.2. Proliferation of Astrocytes in Response to the Exposure of Fluoxetine

**Figure 2:**
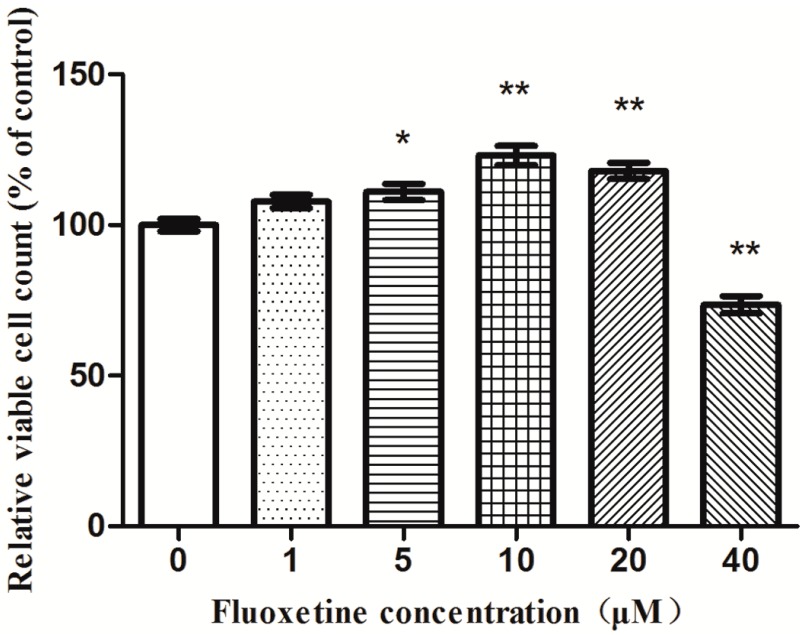
Effects of fluoxetine on astrocytes proliferation. A dose of 10 µM fluoxetine significantly increased astrocyte proliferation relative to untreated controls. Data reported as the means ± SEMs of five independent experiments (*n* = 7). * *p* < 0.05; ** *p* < 0.01, compared with the drug-free controls.

The astrocytes were treated with fluoxetine at various concentrations for 24 h. As shown in Figure 2, fluoxetine at various concentrations from 1 to 20 µM significantly increased the relative numbers of viable astrocytes. Especially at 10 µM, fluoxetine increased the cell number by 23.1%, whereas a 20 µM dose displayed a lower cell number than that of the 10 µM dose. Concentrations greater than 10 µM appeared to produce sub-optimal effects. Thus, the 10 µM dose was chosen for further metabolomics analysis.

### 2.3. ^1^H NMR Spectra

**Figure 3:**
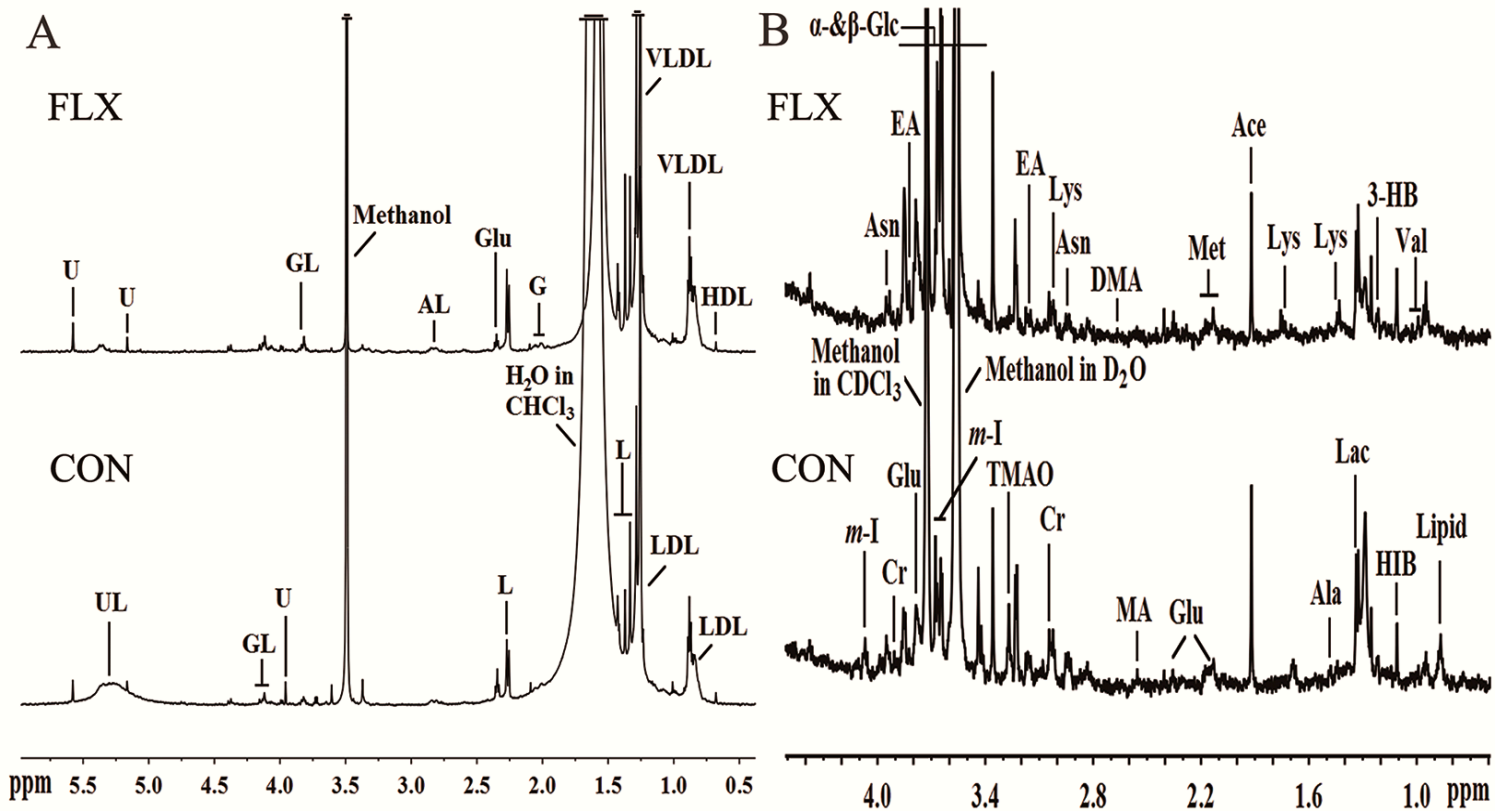
Representative 600-MHz ^1^H NMR spectra of lipid (**A**) and aqueous (**B**) phases of cellular extracts obtained from the control group (CON) and fluoxetine-treated group (FLX). Abbreviations: 3-HB, 3-hydroxybutyrate; Ace, acetate; AL, albumin lysyl; Ala, alanine; Asn, asparagine; Cr, creatine; DMA, dimethylamine; EA, ethanolamine; Fum, fumarate; G, glycoprotein; GL, glyceryl of lipids; Glu, glutamate; HIB, 2-hydrxoyisobutyrate; Ile, isoleucine; L, lipid; Lac, lactate; Leu, leucine; Lys, lysine; MA, methylamine; Met, methionine; m-I, myo-inositol; Suc, succinate; TMAO, trimethylamine N-oxide; Tyr, tyrosine; UL, unsaturated lipid; U, unknown; Val, valine; α-Glc, α-glucose; β-Glc, β-glucose.

Representative 600-MHz one-dimensional Carr-Purcell-Meiboom-Gill (1D-CPMG) ^1^H-NMR spectra obtained from fluoxetine-treated (FLX) and control (CON) cell extracts are shown in Figure 3A,B. In total, 32 unique metabolites were identified including lipid/protein complexes, amino acids, tricarboxylic acid (TCA) intermediates, glucose, waste metabolites and other metabolites.

### 2.4. Multivariate Analysis

**Figure 4:**
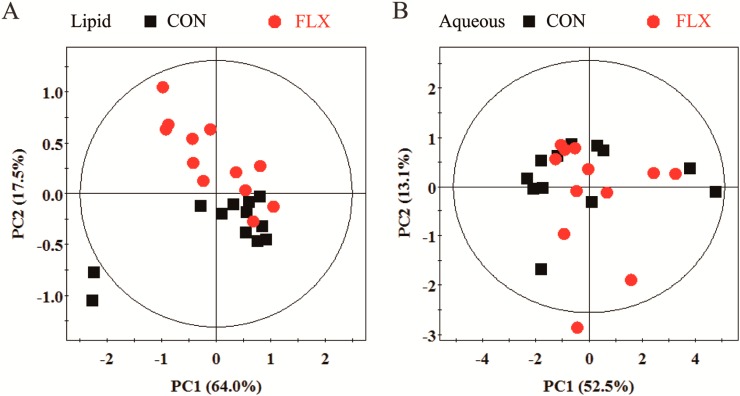
PCA score plot based on ^1^H NMR spectra for the lipid (**A**) and aqueous (**B**) phases of cellular extracts obtained from CON (black box ■) and FLX (red dot ●).

Principal component analysis (PCA) and the findings are displayed in Figure 4A,B. The PCA score plot showed that the two groups overlapped seriously in the aqueous phase or lipid phase. Thus, supervised analysis techniques (partial least-squares discriminant analysis (PLS-DA) and orthogonal partial least-squares discriminant analysis (OPLS-DA)) were then applied to maximize the classification between the two groups.

**Figure 5:**
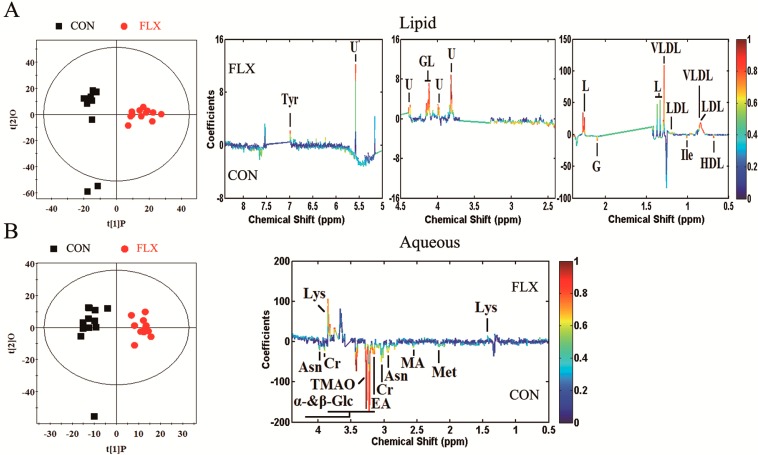
Orthogonal partial least-squares discriminant analysis (OPLS-DA) score plots and corresponding coefficient loading plots. OPLS-DA score plots (**left** panel) and corresponding coefficient loading plots (**right** panel) derived from the ^1^H NMR spectra of the lipid phase (**A**) and aqueous phase (**B**) of cellular extracts obtained from CON and FLX. The color map shows the significance of metabolic variations between the two groups. Positive peaks indicate metabolites that are more abundant in the fluoxetine-treated group, while negative peaks indicate metabolites that are more abundant in the untreated control group.

**Figure 6:**
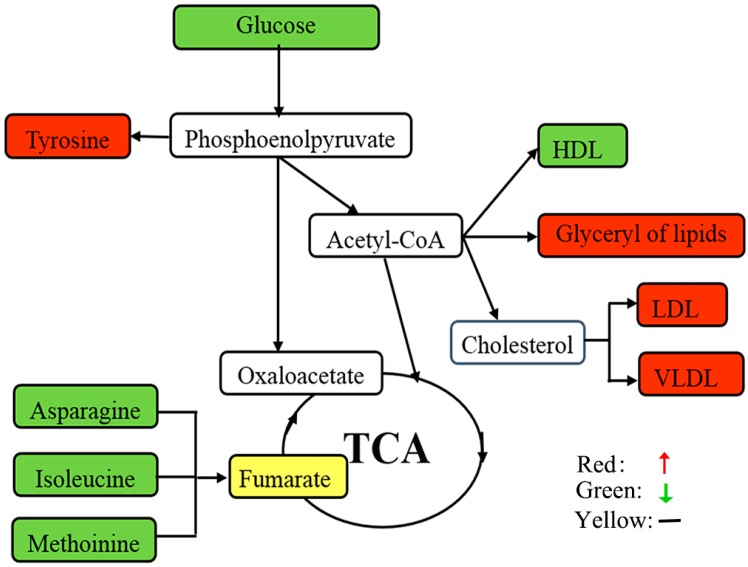
Schematic overview of the metabolite changes in fluoxetine-treated astrocytes. The metabolites are shown in color: red represents increased metabolites; green represents decreased metabolites; yellow represents no change; and the open box represents no detected metabolites. TCA, tricarboxylic acid.

The qualities of the OPLS-DA models were described by the cross-validation parameters Q^2^ and R^2^X. The score plots (R^2^X = 28.0%, Q^2^ = 0.517, lipid phase; R^2^X = 20.2%, Q^2^ = 0.605, aqueous phase) showed clear discrimination between CON and FLX (Figure 5). According to the OPLS-DA analysis, 17 significant differential metabolites were screened out (Table 1). In the fluoxetine-treated samples relative to control samples, the data revealed upregulation of glyceryl of lipid, lipid, low density lipoprotein (LDL), very low density lipoprotein (VLDL), tyrosine and lysine along with downregulation of α-glucose, β-glucose, creatine, glycoprotein, high density lipoprotein (HDL), isoleucine, asparagine, methionine, ethanolamine, methylamine and trimethylamine *n*-oxide (TMAO). The mainly differential metabolites and their respective fold changes were then inputted into the KEGG and Metacore (GeneGo) databases for network analysis (Figure 6).

**Table 1:** Key metabolites discriminating fluoxetine-treated astrocytes and untreated controls in the OPLS-DA model.

Metabolite	Chemical Shift (ppm, Multiplicity)	Metabolic Pathway	r	Change Relative to Control
**Lipid Phase**				
Glyceryl of lipid	4.08(m), 4.13(m), 5.17(s)	Lipid metabolism	0.760	↑
Lipid	1.33(br), 1.37(br), 2.27(m)	Lipid metabolism	0.807	↑
VLDL	0.88(t), 1.29(br)	Lipid metabolism	0.697	↑
LDL	0.84(br), 1.25(br)	Lipid metabolism	0.755	↑
HDL	0.68(br)	Lipid metabolism	−0.675	↓
Glycoprotein	2.10(s)	Amino acid metabolism	−0.793	↓
Isoleucine	1.01(d)	Amino acid metabolism	−0.654	↓
Tyrosine	6.98(d)	Amino acid metabolism, Neurotransmitter metabolism	0.735	↑
**Aqueous Phase**				
α-Glucose	3.44(t), 3.54(dd), 3.71(t), 3.73(m), 3.85(m)	Lipid metabolism-related molecules	−0.684	↓
β-Glucose	3.23(dd), 3.42(t), 3.46(m), 3.49(t), 3.90(dd)	Lipid metabolism-related molecules	−0.964	↓
Creatine	3.04(s), 3.93(s)	Energy metabolism	−0.605	↓
Asparagine	2.94(m), 3.95(m)	Amino acid metabolism	−0.760	↓
Methionine	2.14(s), 3.18(m)	Amino acid metabolism	−0.582	↓
Lysine	1.44(m), 1.75(m), 3.02(m), 3.76(t)	Amino acid metabolism	0.823	↑
Ethanolamine	3.16(t), 3.82(m)	Others	−0.776	↓
Methylamine	2.56(s)	Others, methylamine pathway	−0.583	↓
TMAO	3.27(s)	Others, methylamine pathway	−0.943	↓

Correlation coefficients, positive and negative signs indicating positive and negative correlation in the concentrations, respectively. The correlation coefficient of │r│ > 0.553 was used as the cutoff value for statistical significance based on discrimination significance at the level of *p* = 0.05 and df (degree of freedom) = 11. ‘‘−’’ means the correlation coefficient │r│ < 0.553. Multiplicity: s, singlet; d, doublet; t, triplet; q, quartet; dd, doublet of doublets; m, multiplet.

## 3. Discussion

Fluoxetine has been shown to increase BDNF, VEGF, and VGF expression from cortical astrocytes and promote synaptic plasticity [3,20], factors that exert antidepressant activity [9]. Moreover, glia treatment by fluoxetine has been associated with several metabolic pathways, including energy, glucose and lipid metabolic pathways [3,21,22]. Nevertheless, the molecular basis of fluoxetine treatment upon astrocytes remains largely unknown and requires further elucidation. The aim of this study was to identify differential metabolites in fluoxetine-treated astrocytes *in vitro*.

Through a ^1^H NMR spectroscopy metabolomic approach coupled with OPLS-DA statistical analysis, this study revealed a set of 17 differential metabolites. To develop further insight into the underlying molecular mechanisms at play in astrocytes, these differential metabolites were comprehensively analyzed in terms of metabolic activity. These metabolites were found to be mainly involved in: (i) lipids (apolipoproteins (LDL, VLDL, HDL), glyceryl of lipids, lipid) and lipid metabolism-related molecules (α-glucose, β-glucose, creatine); (ii) amino acids (glycoprotein, isoleucine, tyrosine, asparagine, lysine, methionine); and (iii) other metabolites (ethanolamine, methylamine, TMAO).

### 3.1. Lipids and Lipid Metabolism-Related Molecules

In the present study, levels of the apolipoproteins LDL and VLDL, the glyceryl of lipid and lipids were upregulated in fluoxetine-treated groups *vis-à-vis* untreated controls. In concurrence with previous findings [21,22], these changes suggest that fluoxetine affects lipid metabolic regulation in astrocytes. Apolipoproteins facilitate the transport of various lipid molecules, including cholesterol, triglycerides and glycosphingolipids [23]. Previous studies by our research group and others have revealed an association between cholesterol level and depression [2,10,24,25]. Since the CNS is separated from the systemic circulation by the blood-brain barrier (BBB), numerous observations uncovered that astrocytes must serve as sources of cholesterol for neurons [26,27] and oligodendrocytes [28,29]. Therefore, as cholesterol is an essential component in synaptogenesis and myelin growth, the formation, function and stability of synapses would be particularly sensitive to the disturbances in cholesterol metabolism within astrocytes [29,30]. Additionally, fluoxetine therapy has been reported to affect weight gain [31] and activate SREBP transcription factors to induce cholesterol and fatty acid biosynthetic pathways in cultured human glial cells [21]. Moreover, as important components of cell membranes, the upregulation of lipids provides important materials for cell proliferation, which supports the findings from our cell proliferation assays previously mentioned.

Furthermore, several lipid metabolism-related molecules, namely α-glucose, β-glucose and creatine, were also differentially identified in fluoxetine-treated astrocytes *vis-à-vis* untreated controls. Specifically, the fluoxetine-treated group displayed lower glucose levels, which might promote lipogenesis and increase cholesterol levels in astrocytes. Consistent with our findings, previous analysis revealed that fluoxetine reduced the glycogen level, increased glucose utilization and promoted lactate release in astrocytes [3].

Based on the foregoing data and analysis, lipids and glucose levels most clearly distinguished the two groups. Considering that all of the molecules were central to lipid metabolic regulation, the possible contribution of fluoxetine was, by increasing the lipids and cholesterol expression from astrocytes, to normalize the trophic and metabolic support to neurons and oligodendrocytes in depression.

### 3.2. Amino Acid Metabolism

Glycoprotein and several amino acids were also differentially identified in fluoxetine-treated astrocytes relative to untreated controls. Specifically, we observed decreases in glycoprotein and three amino acids (*i.e*., isoleucine, asparagine and methionine) accompanied by increases in two amino acids (*i.e*., lysine and tyrosine) in fluoxetine-treated astrocytes. Amino acid metabolism is complicated, as proteolysis, gluconeogenesis and oxidative catabolism all contribute to amino acid homeostasis [23]. Notably, all of the downregulated amino acids are involved in gluconeogenesis in astrocytes [32]. As important gluconeogenesis precursors, these amino acid levels may decrease as a response to increased glucose utilization, thus ensuring the normal supply of energy. This hypothesis is consistent with the fact that the TCA cycle intermediates were not significantly perturbed in fluoxetine-treated astrocytes *vis-à-vis* untreated controls.

In addition, lysine levels were substantially increased in fluoxetine-treated astrocytes relative to untreated controls. Dietary lysine deficiency has been demonstrated to increase stress-induced anxiety in rats [13,33], while lysine fortification has been shown to reduce anxiety in humans [14]. Meanwhile, lysine has been shown to act as a serotonin receptor 4 (5-HT4) antagonist and is effective in treating animal models of serotonin (5-HT)-induced anxiety [34]. Therefore, the observed lysine upregulation in fluoxetine-treated astrocytes suggests that fluoxetine therapy positively affects lysine levels in astrocytes, which may affect 5-HT-induced affective states.

Furthermore, tyrosine levels were substantially increased in fluoxetine-treated astrocytes relative to untreated controls. Consistent with this finding, our lab’s previous study revealed tyrosine downregulation in the brain of a chronic unpredictable mild stress (CUMS) rat model of depression [11]. Moreover, perturbation of tyrosine levels has also been observed in a CUMS rat model of depression and depressed human patients [12,35]. Therefore, the observed tyrosine upregulation in fluoxetine-treated astrocytes suggests that fluoxetine therapy may modulate mood through a tyrosine-associated mechanism.

However, the results and conclusions of this study should be cautiously interpreted because of several limitations. Firstly, the sample size was relatively small, so further studies with larger cohorts should be performed to validate our findings. Secondly, the current data were obtained from intracellular metabolites *in vitro*, so future studies should be investigated by collecting and analyzing extracellular fluid and animal experiments *in vivo*. Finally, owing to the diverse physicochemical properties and wide concentration range of metabolites, additional metabolomic methods or multiple metabolomic platforms (*i.e*., stable isotope-resolved metabolomic analysis, GC-MS, LC-MS, target metabolomics) should be employed in future studies [36,37,38,39].

Specifically, to further study the potential features of metabolism and interactions between neurons and glia in the fluoxetine-treated model, we have planned to investigate the relative importance of lipids and cholesterol using ^13^C-labeled tracers and ^13^C isotopomer-resolved metabolomic (SIRM) analysis by NMR and LC/GC-MS, which could clearly show the flow of metabolites between astrocytes and neurons [36,37].

## 4. Experimental Section

### 4.1. Materials

Dulbecco’s Modified Eagle’s Medium (DMEM), fetal bovine serum (FBS), phosphate-buffered saline (PBS) and 0.25% trypsin-EDTA were purchased from Hyclone, Beijing, China. Cell culture dishes, flasks and 96-well plates were purchased from Corning, New York, NY, USA. Ultrapure water was prepared through the Millipore MilliQ purification system. Analytical-grade methanol, chloroform, Deuterium oxide (D_2_O) containing 0.05 wt % 3-(trimethylsilylpropionic-2,2,3,3,-d 4 acid sodium salt, TSP), poly-d-lysine and fluoxetine were purchased from Sigma-Aldrich, New York, NY, USA. The Cell Count Kit-8 (CCK-8) assay was purchased from Beyotime, Jiangsu, China. The GFAP antibody and the goat anti-rabbit IgG Alexa Fluor^®^ 488-conjugated antibody were obtained from Invitrogen, New York, NY, USA.

### 4.2. Primary Culture of Cortical Astrocyte

Sprague-Dawley rats (1–2 days old) were obtained from Chongqing Medical University’s animal center (Chongqing, China). Primary astrocytes were prepared as described previously [3] with minor modifications. Briefly, cells were obtained from the cerebral cortices of rats and seeded on T75 flasks (coated with poly-d-lysine) in DMEM supplemented with 15% FBS. Culture medium was changed every three days until cells reached confluence after about 10 days. Then, astrocytes were separated from contaminating microglia and oligodendrocytes through shaking and mild trypsinization and then grown in new coated flasks. All experiments were performed using cells from the third passage that were highly enriched in astrocytes (identified by >95% purity of GFAP, an astrocytic protein marker).

### 4.3. Immunofluorescence

Cells on glass cover slips were fixed with 4% paraformaldehyde, rinsed with PBS, incubated with 0.3% TritonX-100 for 5 min and blocked in 3% BSA for 60 min. The cover slips were then incubated in primary antibody overnight at 4 °C, washed with PBS and incubated with fluorescence-conjugated secondary antibody at 37 °C for two hours. The cover slips were washed with PBS three times, then mounted with mounting medium containing DAPI (4',6-diamidino-2-phenylindole) and examined by fluorescence microscopy (Nikon, Tokyo, Japan).

### 4.4. Stimulation and Proliferation Assays

Cell proliferation was determined using the CCK-8 assay according to the manufacturer’s instructions. Astrocytes were plated at a density of 5 × 10^3^ cells/well into coated 96-well plates in 100 μL culture medium. When cells reached 70%–80% confluence, the culture medium was removed, and astrocytes were incubated in serum-free DMEM supplemented with various concentrations (0, 1, 5, 10, 20 or 40 μM) of fluoxetine for 24 h. Next, 10 μL CCK-8 reagent were added to each well and incubated for 2 h at the end of the 24-h incubation period. The absorbance of the colored solution was measured using an enzyme-linked immunosorbent assay reader (Bio-Rad, Hercules, CA, USA) (λ = 450 nm).

### 4.5. ^1^H NMR Sample Preparation

Astrocytes were cultured at a density of 2 × 10^6^ cells/dish in 24 separate coated 100-mm dishes. Twelve dishes were exposed to fluoxetine with a concentration of 10 μM, a highly effective concentration selected by the proliferation assay. The remaining 12 control dishes were treated with only serum-free DMEM. After 24 h, the extraction procedure was performed on a crushed ice bath (4 °C) according to a modified method as previously reported [19,40]. Cells were trypsinized using trypsin/EDTA solution and washed in PBS, followed by being pelleted into a 1.5-mL Eppendorf tube.

Analytical-grade methanol and chloroform in a 2:1 ratio (*v*/*v*; 500 µL/cell pellet) at 4 °C were added to the cell pellets. The pellet was resuspended using a vortex mixer (Sonics, Newtown, CT, USA). Then, the cell pellet-solvent mixture was sonicated. After approximately 15 min with the first solvents, chloroform and distilled water were added to the samples in a 1:1 ratio (500 µL/cell pellet) and mixed again. The samples were then centrifuged at 10,000 rpm for 15 min to allow clear separation between the aqueous and lipophilic layers. The two layers were separated and freeze-dried. Aqueous portions were reconstituted with 500 μL phosphate buffered D_2_O (containing 0.05% TSP) at pH 7, and lipophilic portions were reconstituted with 500 μL deuterated chloroform. The reconstituted samples were pipetted into 5-mm NMR tubes for ^1^H NMR experiments [19].

### 4.6. ^1^H NMR Spectroscopy

^1^H NMR spectra for all extracts (hydrophilic and lipophilic) were collected on the Varian Unity INOVA 600 NMR spectrometer (Varian Inc., Palo Alto, CA, USA), at a frequency of 599.925 MHz. Aqueous samples were analyzed using a conventional pre-saturation pulse sequence with solvent suppression NOESYPR1D (RD-90°–t1-90°–tm-90°–ACQ) (relaxation delay = 2.5 s, mixing time = 0.1 s; solvent pre-saturation was applied during the relaxation delay and mixing time). Lipophilic samples were analyzed similarly, but without solvent suppression.

The NMR data for cell extracts were converted into Bruker format from Varian format before data processing. For all 1D ^1^H NMR spectra, free induction decays (FIDs) were multiplied by an exponential function with a 1.0-Hz line-broadening factor prior to Fourier transformation. All of the ^1^H NMR spectra were manually phased and baseline-corrected using Topspin 3.0 software (Bruker Biospin, Karlsruhe, Germany). Aqueous sample spectra were normalized to the TSP peak at δ0.00, and lipophilic sample spectra were normalized to the CHCl_3_ peak at δ7.26. The spectral regions δ8.50–0.50 (for the lipophilic samples) or δ4.40–0.50 (for the aqueous samples) were automatically divided into integral segments of equal width (0.002 ppm) using AMIX (V3.9, Bruker Biospin, Karlsruhe, Germany). For the NMR spectra of the lipophilic portions, the regions of δ7.50–7.00, δ5.15–4.40 and δ2.05–1.43, δ3.70–3.30 were excluded to eliminate the CHCl_3_, residual water and ethanol signals, respectively. For the NMR spectra of the aqueous portions, the regions of δ3.75–3.70, δ3.59–3.51 and δ3.33–3.37 were excluded to eliminate the impurity and ethanol signals, respectively. The integrated data were normalized to the total sum of the spectrum before multivariate statistical analysis to give the same total integration value for each spectrum before data analysis.

### 4.7. Multivariate Data Analysis

Multivariate statistical analysis was carried out using the SIMCA-P+ software package (V11.0, Umetrics AB, Umea, Sweden). Principle component analysis (PCA), performed by using a mean-centered scaling approach, was initially applied to the spectral data to visualize inherent clustering between the control group and fluoxetine-treated group. After the overview of the NMR data using PCA analysis, the data were subjected to OPLS-DA, and a model was built and utilized to identify marker metabolites that accounted for the differentiation between the two groups [41].

OPLS-DA as an extension of partial least-squares discriminant analysis (PLS-DA) featuring an integrated orthogonal signal correction (OSC) can remove variability not relevant to class separation. Both PLS-DA and OPLS-DA were based on a unit variance scaling strategy. The validation of the model was conducted using 6-fold cross-validation, with the cross-validation parameters R^2^ and Q^2^ representing the predictive ability of the model and the explained variance, respectively. To further validate the quality of the PLS-DA models, permutation tests consisting of a randomly permuting class membership with 200 iterations were carried out. The sensitivity, specificity and classification rate of OPLS-DA models were then depicted.

### 4.8. Statistical Analysis

Data of the proliferation characteristics were presented as the means ± SEMs and analyzed by one-way ANOVA followed by Tukey’s *post hoc* test. Statistical analyses were carried out with SPSS software (version 21.0, IBM, Chicago, IL, USA). *p* < 0.05 was considered as significant.

## 5. Conclusions

In conclusion, this is the first study to apply a ^1^H NMR metabolomic approach to identify differential metabolic profiles in fluoxetine-treated astrocytes. Comparative metabolomic profiling reveals significant alterations in levels of lipids, lipid metabolism-related molecules and amino acids in fluoxetine-treated astrocytes. Thus, fluoxetine may normalize astrocytic regulation of lipid and cholesterol metabolism, thus providing the trophic and metabolic support for neurons and oligodendrocytes in depressed patients. Moreover, fluoxetine therapy appears to enhance lysine and tyrosine levels, which may contribute to its antidepressant effects. Through elucidating the intracellular metabolic changes incident to fluoxetine treatment *in vitro*, this study also provides direction for future exploration on the effects of fluoxetine exposure in extracellular fluid and *in vivo*.
